# Molecular Phenomic Approaches to Deconvolving the Systemic Effects of SARS-CoV-2 Infection and Post-acute COVID-19 Syndrome

**DOI:** 10.1007/s43657-021-00020-3

**Published:** 2021-07-22

**Authors:** Jeremy K. Nicholson

**Affiliations:** 1 The Australian National Phenome Center and the Health Futures Institute, Murdoch University, Harry Perkins Building, Robert Warren Drive, Murdoch Perth, WA 6150 Australia; 2 Institute of Global Health Innovation, Imperial College London, Level 1, Faculty Building South Kensington Campus, London, SW7 2NA UK

**Keywords:** SARS COV-2, COVID-19, Post-acute COVID-19 Syndrome (PACS), Spectroscopy, Metabolic Phenoconversion, Phenoreversion

## Abstract

SARS COV-2 infection causes acute and frequently severe respiratory disease with associated multi-organ damage and systemic disturbances in many biochemical pathways. Metabolic phenotyping provides deep insights into the complex immunopathological problems that drive the resulting COVID-19 disease and is also a source of novel metrics for assessing patient recovery. A multiplatform metabolic phenotyping approach to studying the pathology and systemic metabolic sequelae of COVID-19 is considered here, together with a framework for assessing post-acute COVID-19 Syndrome (PACS) that is a major long-term health consequence for many patients. The sudden emergence of the disease presents a biological discovery challenge as we try to understand the pathological mechanisms of the disease and develop effective mitigation strategies. This requires technologies to measure objectively the extent and sub-phenotypes of the disease at the molecular level. Spectroscopic methods can reveal metabolic sub-phenotypes and new biomarkers that can be monitored during the acute disease phase and beyond. This approach is scalable and translatable to other pathologies and provides as an exemplar strategy for the investigation of other emergent zoonotic diseases with complex immunological drivers, multi-system involvements and diverse persistent symptoms.

## Global Challenges of the COVID-19 Pandemic and Post-acute COVID-19 Syndrome

The exceptionally rapid local and international spread of COVID-19 has posed a uniquely dynamic and interrelated set of medical and economic problems that have directly or indirectly affected the lives of most of the world’s population. Global healthcare and disease burden “fall-outs” of COVID-19 will also have significant global societal impacts for many years to come. Climate change, the increase in human population, and changes in geographical population distributions have increasingly brought humans and wild animals together in ways that enable and assist the transmission of novel zoonotic diseases to man (Bartlow et al. 2019). The SARS CoV-2 b-coronavirus is one of a series of emergent zoonotic threats, and it was inevitable that a spill-over into man would occur from one of these pathogens and provoke a medical disaster. The speed of SARS CoV-2 spread was facilitated by a combination of modern human social behaviours, globalization of business practises, the commonality of long-distance travel, and high-density population packing that enhances the transmissibility of any infectious disease. SARS CoV-2 has proved to be a particularly dangerous enemy because of its mode of airborne transmission and infectivity from people with mild or non-obvious symptoms. The virus is also evolving, and multiple variants are driving new waves of the pandemic (Lotfi et al. 2020; Van Vinh Chau et al. 2020). The direct economic coupling of the disease pandemic has also changed the world and human societal relationships irreversibly, and this continues to raise international political tensions. The global propagation of this dangerous and poorly understood pathogen has challenged individuals, populations, businesses, as well as scientists and doctors in ways that were scarcely imaginable only a year or two ago.

Within a few months of the first COVID-19 outbreaks, some populations were forced into living conditions more reminiscent of wartime. The rate of disease spread posed immediate challenges to medical capacities and capabilities in many countries, and there was a medical, social, and political need to explain what is COVID-19, how it is transmitted, its effects and how to prevent, detect and treat it? Many of these questions have now been at least partially answered due to the huge international research efforts involved, and we also know some of the major predisposing risk factors for severe disease such as body mass index, background pulmonary disease, and diabetes (Van Vinh Chau et al. 2020). There are still major gaps in our mechanistic understanding of the disease process and the highly complex systemic dysfunction that leads to persistent symptoms and long-term damage associated with PACS (Nalbandian et al. 2021). But COVID-19 is also a major social problem and the ability to deploy new knowledge to affect clinical translation are being confounded by political indecision and population complacency, even in countries that have had success in managing patient numbers down through lockdowns and vaccinations. New SARS COV-2 variants with higher receptor binding and higher infectivity such has B.1.1.7 (Collier et al. 2021) and more recently B.1.617.2 are also taking advantage of multiple gaps in our defences and collective poor behaviours and are driving new outbreaks.

Paradoxically, some of the greatest advances in science and engineering occur in times of war, when there is a technical race is to create new defences, weapons and strategies. In the present case of COVID-19, there is a global war against an unseen and adaptable enemy, the coronavirus, and that has resulted in major advances in medical technologies. These advances will be put to good use in future against other zoonotic viral enemies. The extraordinary scientific progress in understanding the disease, and the creation of new vaccines in record time (normally years of research and development compressed into months) has been remarkable, and this is a clear sign of hope for the implementation of practical disease mitigation strategies in the future. The pandemic also revealed profound weaknesses in societal preparedness, political decision-making, and some antisocial human behaviours that helped the virus propagate with multiple outbreak waves that cannot be managed by scientific methods alone, and those problems still pose significant threats to the worldwide control of the virus. We now face the emerging and largely unknown challenges of PACS, which may ultimately affect millions of people, and there is an urgent need to develop new tools for measuring, monitoring, and mitigating persistent clinical symptoms and systemic disease consequences of SARS CoV-2 infection.

## The Acute and Chronic Faces of COVID-19

The acute effects and progression of COVID-19 have now been well-documented in multiple populations. As a new type of b-coronavirus with largely airborne transmission, the most common severe and life-threatening effects are respiratory. Disease severity and respiratory failure are often exacerbated by underlying chronic disease, such as diabetes and these are more likely to be present in elderly patients, but young people can also be severely affected (Lotfi et al. 2020; Van Vinh Chau et al. 2020). However, other systemic effects of COVID-19 have also appeared and form a common pattern of the disease which can be present even in the absence of severe respiratory symptoms. The systemic immunopathological responses to SARS CoV-2 infection themselves affect multiple organs and some of these are similar to the effects of related coronaviruses that caused Middle East Respiratory Syndrome (MERS) and Severe Acute Respiratory Syndrome (SARS). COVID-19, like MERS and SARS also causes significant multi-system long-term effects (Ahmed et al. 2020; Cortinovis et al. 2021; Wang et al. 2020) including liver dysfunction, kidney dysfunction, cardiovascular disease, anosmia and neurological problems, and a host of other rarer complications (Wang et al. 2020; Wu et al. 2020a). Many COVID-19 patients do not readily recover from these ailments, and a high proportion of patients, are still multi-symptomatic with PACS months, and possibly years, after the acute phase with reports of long-term pulmonary, hematologic, cardiovascular, renal, neuropsychiatric, endocrine, gastrointestinal, hepatobiliary, and dermatological problems PACS patients (Nalbandian et al. 2021).

## Why Molecular Phenomics is a Powerful Tool for Understanding COVID-19?

Phenomics is the systematic study of the continuum of gene-environment interactions that occur throughout life, and the measurement of the emergent physical and chemical properties that result from those interactions that define individual and population phenotypes in health and disease. These same combinations of interactions give rise to a variety of disease risks in individuals populations and help define the phenomic properties of disease states that can be applied to diagnose and stratify patients (Nicholson 2006; Holmes et al. 2008a; Nicholson et al. 2002). In *molecular phenomics,* we are concerned with the chemical and biochemical signatures (metabolites, proteins, transcripts, etc.) of cells and biofluids, and how these change in characteristic ways during the onset, development, and recovery from disease. This type of information is essential to the molecular characterization of emergent diseases such as COVID-19, and to understand their systemic effects. Such molecular characterization requires the use of comprehensive multiparametric measurements that in the case of blood chemistry, proteins, and metabolites is the domain of mass spectrometry and NMR spectroscopy, which form the main basis of this discussion. Metabolic phenotyping or Metabotyping (Gavaghan et al. 2000) is a subclass of metabolic research that is closely aligned with metabonomics and metabolomics in which multiple metabolites representing many pathways and chemical classes are measured to give a global representation of ongoing systemic disease processes or the patho-physiological status of the individual (Nicholson et al. 2012).

Understanding any emergent disease is both a clinical and molecular discovery process, and so we seek to measure many biochemical components in multiple pathways that relate to the clinical signs and symptoms and the whole-body responses to the disease development through time. Metabolic frameworks for understanding such processes have been described previously (Nicholson et al. 2012). The emphasis being on obtaining significant metabolic pathway coverage and hence the application of multiple techniques in parallel to explore many biochemical features of the disease in individual patients. Ideally, the studies are performed wherever possible on samples from the same patients, because this enables the use of statistical spectroscopy and other data fusion tools to construct integrated multivariate models describing the *integrated* systemic responses to disease. This also paves the way to the discovery of new biomarkers relating to diagnosis and prognosis of the disease as well as monitoring progression and recovery processes (Nicholson et al. 2012).

## The Metabolic Continuum from Health to COVID-19 Disease Development

**Fig. 1:**
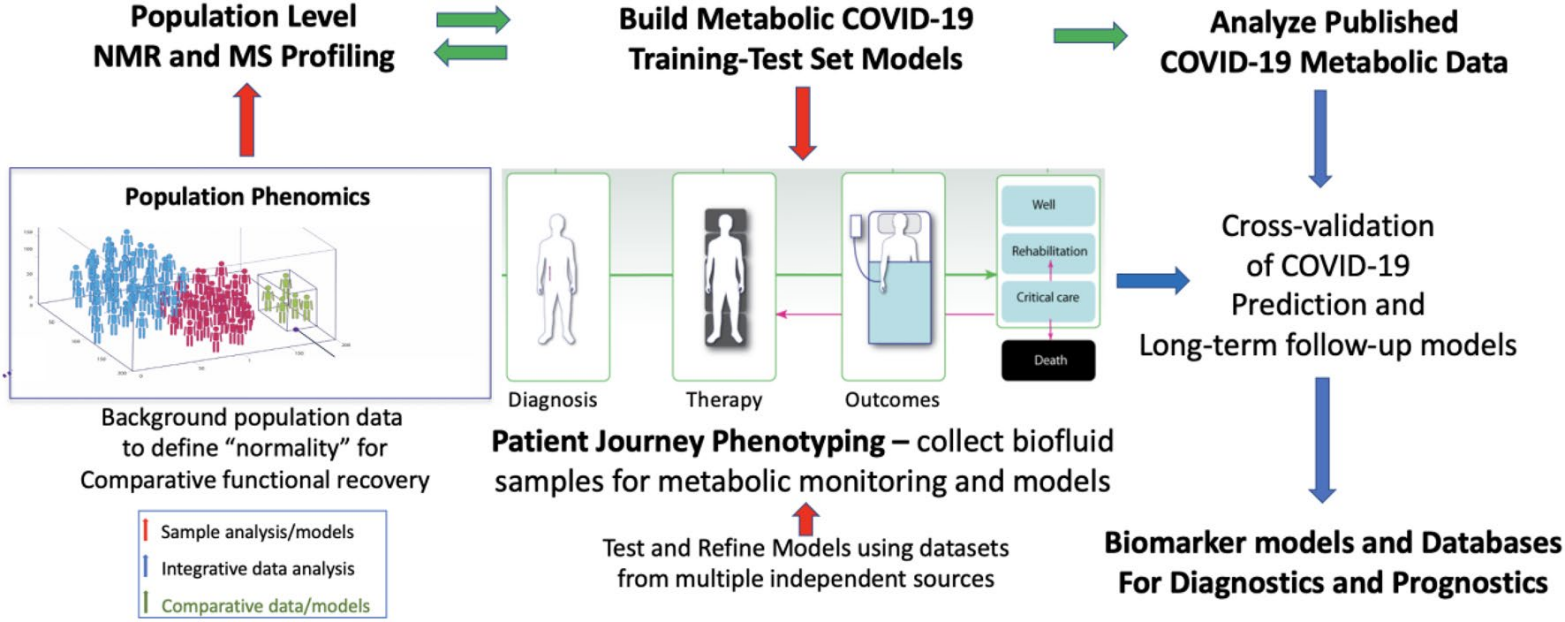
A framework for understanding the natural history of an emergent zoonotic disease using metabotyping: schematic illustration of the collective COVID-19 patient journey from health to disease using a metabolic systems framework to assess disease progression and recovery in relation to associated studies that enable model cross-validation through published literature and sequential analysis of multiple disease cohort samples. The population phenomics box illustrates the collection of different metabolic signatures from population subgroups some of which may have different disease risks

The initiation of a disease process in a healthy individual involves either a series of complex gene interactions over long periods of time or an initial acute event, such as an infection, that rapidly disrupts homeostasis and changes the function and phenotype of the organism. The degree (severity) and rates of such molecular phenotypic changes are also dependent on the history of the individuals’ gene-environment interactions as well as age, gender, ethnicity, nutrition, immune status and the presence of confounding disease. This can be thought of as an extension of the *patient journey concept* in which the metabolic trajectory of individuals is measured and monitored through time to augment understanding of the systemic responses to therapy (Nicholson et al. 2002, 2012). A summary of the extended concept of the metabolic journey from health to disease and recovery is provided in Fig. 1.

To understand the metabolic starting properties of a population group, there should be somebody of reference data and materials to define “normality” for the age, gender, and ethnicity distribution of the population. Such information can often be obtained from appropriate screening of epidemiological samples (Holmes et al. 2008a, b), and this also serves as a reference framework for assessing functional recovery from a disease process by comparing the patients in the post-acute disease phase with data from their own relevant pre-disease population group.

The patient journey phenotyping process involves the discovery of new metrics that help quantify and map disease progression in a metabolic framework and can lead to new diagnostic and prognostic biomarker discoveries. The aim of this approach is to cross-model and cross-validate biomarkers derived from the molecular study of multiple patient journeys from many sources. This process results in new molecular insights into the systemic disease process and the possibility of development of metrics for studying functional recovery from the disease. The approach is illustrated for COVID-19 Fig. 1 but, in principle, the concept is scalable and translatable to any emergent disease, especially those with multiple systemic involvements such as COVID-19 (Gavaghan et al. 2000; Nicholson et al. 2012; Holmes et al. 2008b; Kimhofer et al. 2020).

## What can Immuno-metabolic Phenotyping Teach us About COVID-19?

Metabolic phenotyping provides comprehensive broad systems level information that can be directly related to pathway and organ dysfunction, so it is an intrinsically translational -omics science. COVID-19 manifests itself as a multi-organ and multi-system disease with a complex presentation of symptoms, target organs and biochemical perturbations. It is essentially a systemic metabolic disease driven by the heightened activities of the immune system responses to the viral presence. Profound effects are seen in lipid (Kimhofer et al. 2020; Chen et al. 2020) and amino acid (Wu et al. 2020b; Cai et al. 2020; Lawler et al. 2021) metabolism and other small molecules as well as shifts in lipoprotein profiles, these changes reflecting liver, cardiovascular, diabetic, muscle wasting, and neuropathological effects (Kimhofer et al. 2020; Chen et al. 2020; Wu et al. 2020b; Cai et al. 2020; Lawler et al. 2021; Lodge et al. 2021a). There is also a plethora of other effects including anosmia, Guillain–Barre syndrome, and chronic fatigue) and most of those symptoms can be persistent in patients with PACS (Nalbandian et al. 2021).

We have referred previously to COVID-19 as a *mosaic disease* that we are attempting to understand from a fragmented pattern of signs, symptoms, and chemical pathologies to enable improved patient management (Kimhofer et al. 2020). The complex shifts in metabolism observed in COVID-19 are not uniformly expressed, nor are they necessarily proportional to disease severity as simply classified by respiratory symptoms, although many changes are proportional to the lung damage which in its severe form is primarily immunologically driven. Indeed, the early immune responses are major determinants of individual clinical outcomes (Bergamaschi et al. 2021). Recent immunological findings suggest that there might be limitations in the clinical value of early stage severity prediction because early CD8+ bystander cytotoxic T cell responses are responsible for the mitigation of severe lung injury and patients only have mild symptoms. Patients that fail to show strong CD8+ response appear to progress rapidly to severe respiratory disease, their disease trajectory having already have been set by the time of initial presentation at the clinic (Bergamaschi et al. 2021). Given the narrow time windows involved for such severity progressions, which can be as little as a few hours from initial hospital presentation to critical care hospitalisation, there is insufficient time to test and intervene to modify the acute disease outcome. Nevertheless, early metabolic, and immunological responses may be predictive of PACS. This may provide a more clinically translatable and tractable metabolic modelling approach as the evolution of PACS takes place over weeks to months rather than hours to days as in the acute disease phase, however, this is still untested.

## Analytical Considerations, Windows on Metabolism, and Biochemical Findings in COVID-19 Research

Several powerful structural analytical and quantitative measurement tools are available to study typical patient samples that contain systemic metabolic information. The most notable of these are Nuclear Magnetic Resonance (NMR) spectroscopy and mass spectrometry (MS) and these can readily be applied to molecular phenotyping of biofluid samples (Nicholson et al. 2012). However, some early studies on the metabolic sequelae of COVID-19 were poorly sampled and performed with inappropriate sample preparation, partially driven by caution concerning the handling of samples that potentially contained live virus in analytical laboratories without appropriate microbiological physical containment capabilities. Methods involving sample heating (typically at 56 °C for 30 min) were employed to inactivate the virus. Unfortunately, this process irrevocably distorts the molecular composition of blood plasma leading to the loss of biomarker information via denaturation or chemical hydrolysis, or worse the physical or chemical creation of false *pseudo*-markers that are products of the heat treatment process rather than the disease itself (Loo et al. 2020).

The various spectroscopic “windows” into the disease process reveal different, but complementary, insights into the altered biochemistry and pathological processes. NMR spectroscopy of blood plasma has given new metabolic, glycoproteomic and lipoproteomic insights into the acute COVID-19 disease process and the relationships between these parameters and cytokines and chemokines indicates the presence of an activated immune-metabolic axis (Kimhofer et al. 2020; Lawler et al. 2021; Lodge et al. 2021a). Even simple metabolic measurements can be revealing, thus, in severely affected patients, the plasma lactate to pyruvate ratio can be high reflecting impaired peripheral blood oxygenation and poor pulmonary function (Kimhofer et al. 2020). More complex measurements enable deeper understanding of immune-metabolic interactions that underpin the disease. Proton NMR methods allow simultaneous observation of metabolites, lipoproteins and glycoproteins (Nicholson et al. 2002, 2012). Thus, acute-phase reactive proteins such as a-1-acid glycoprotein (GlycA) are highly elevated in plasma in active COVID-19 disease, and there is also a marked reduction in HDL-related species, e.g., phospholipids, with a significant rise in many VLDL and LDL components (Kimhofer et al. 2020; Lodge et al. 2021a). In particular, the Apolipoprotein B100/Apolipoprotein A1 ratio (ABA1) is increased in active COVID-19 disease and ABA1 values have previously been associated with increased cardiovascular and atherosclerotic risks (Holmes et al. 2008b; Lodge et al. 2021a). Mass spectrometry (MS) is also contributing significantly to the understanding of perturbed COVID-19 biochemistry and MS metabolic windows give highly complementary metabolic information to the NMR windows. Thus, disturbance of the tryptophan-kynurenine pathway (as measured by mass spectrometry, 20) is also associated with atherogenic risk (Wang et al. 2015). Tryptophan/kynurenine pathway disruption is a feature of many acute inflammatory conditions (Chen and Guillemin 2009; Lovelace et al. 2017; Thomas et al. 2020), all via immunologically driven cellular processes involving stimulation by interferon-g, TNF-a and other cytokines and chemokines (Lawler et al. 2021). The disturbances to this pathway in COVID-19 as measured by MS of blood plasma are profound, with elevated quinolinic acid and 3-hydroxykynurenine and kynurenine together with reduced tryptophan (Lawler et al. 2021) providing an additional atherogenic driver over and above the atherogenic lipoproteins profile, particularly as reflected in the plasma ABA1 ratio. The plasma tryptophan-kynurenine ratio is high during active COVID-19, and it is also elevated in a variety of neurological/neuro-immune disorders (Lovelace et al. 2017). Indeed, SARS COV-2 infection also causes multiple neurological side-effects and manifestations which can be long-lasting (Varatharaj 2020), and it is likely that these relate to the tryptophan-kynurenine pathway as part of the multi-level systems involvement in the complex of acute COVID-19 pathologies. Thus, individual metabolic pathway abnormalities (such as tryptophan-kynurenine) contribute to the development of disparate pathologies such as the cardiovascular and neurological effects seen in COVID-19.

Mass spectrometry is also contributing to understanding the systemic effects of COVID-19 on lipid metabolism. For instance, there are major shifts in the multiple lipidic pathways and concentrations but these lipidomic signatures are still poorly understood from a mechanistic viewpoint and are the subject of intense study. A recent untargeted metabolic and lipidomic study uncovered multiple potential diagnostic and mechanistic biomarkers including phosphatidylcholines, phosphatidylethanolamines, triglycerides, free fatty acids (especially arachidonic acid and oleic acid), porphyrins, and aromatic amino acids were identified as being diagnostic (Bruzzone et al. 2020). Pathway enrichment analysis revealed several candidate mechanistic possibilities, but interpretation of such data is constrained by the breadth of the pathway input information, which equates the number and diversity of the metabolites measured analytically. So, the multiple window approach is necessary to examine interrelated pathological drivers from multiple compartments. Ultimately, untargeted analysis requires quantitative cross-validation using isotope dilution tandem mass spectrometry for instance to establish robust quantitative biomarker ranges that are required for real-world clinical deployment. Furthermore, many of the observed cellular and systemic metabolic disruptions are modulated by cytokine activities (Lawler et al. 2021; Lodge et al. 2021a) that need to be measured and co-modelled to gain deeper mechanistic insights.

Novel NMR spectroscopic methods utilising combined relaxation and diffusion editing have identified new and highly diagnostic molecular biomarkers in specific molecular compartments that have specific motional properties. These methods can be used to distinguish COVID-19 from other mild respiratory diseases with high fidelity based on immune-metabolic signatures of supramolecular lipoprotein and glycoprotein complexes (Lodge et al. 2021b). This work led to the discovery of a novel class of supramolecular structure composite (SPC) NMR signal markers which are characteristically reduced in COVID-19. These signals originating from phospholipids, especially lysophosphatidylcholines present mainly in lipoprotein HDL sub-compartments or bound to glycoproteins. The immunopathological significance of these signals still remains to be discovered but the SPC/GlycA ratio is highly discriminating between SARS COV-2 positive patients and others with mild respiratory disease awaiting diagnosis (Lodge et al. 2021b).

There has been a rapid proliferation of COVID-19-related metabolic literature from many laboratories using a variety of analytical instrumentation, different patient cohorts from different genetic backgrounds, with varying severities and time sampling points. Harmonisation of technology and approach in phenomic analytical chemistry has been a long-term objective in the metabolic phenotyping field (Lindon et al. 2005). In the case of COVID-19 metabolic studies, standardization, and cross-validation of sample preparation methods has been investigated and recommendations made to ensure robust metabolic data collection that enable interlaboratory comparisons (Loo et al. 2020; Meoni et al. 2021). In the case of NMR spectroscopy, protocol harmonization already exists for in vitro diagnostic applications such as plasma metabolites and lipoproteins (Meoni et al. 2021) and this has led to highly complementary analytical data being generated in multiple laboratories studying COVID-19 round the world (Jimenez et al. 2018; Barberis et al. 2020) which gives confidence that these biomarker signatures of the disease are accurate and translatable across populations.

## Systemic Post-acute COVID-19 Syndrome, Phenoreversion, and Patient Recovery

It is notable, that although the actual disease presentation and the clinical severity classifications are dominated by the pulmonary pathology, respiratory symptoms are just “the tip of the iceberg” in terms of the underlying biochemical and immunological dysfunction caused by SARS CoV-2 infection. From a clinical viewpoint, the systemic nature of COVID-19 needs to be considered carefully with respect to both the patient severity classification and what the different systemic dysfunctional expression means in relation to physical patient assessment, management and long-term therapy. The deep metabolic perturbations caused by the virus are also relevant for our understanding of the COVID-19 “recovery” process and the development of new metrics for assessment of PACS at the personalized level. An individual can only be thought of as “fully recovered “when their biochemical profiles are substantially normalized with respect to the earlier systemic perturbations seen in the acute phase. This process of normalization is effectively the opposite of disease phenoconversion (Kimhofer et al. 2020) and we refer to this process as *Phenoreversion* and this can be measured metabolically (Holmes et al. 2021). Phenoreversion appears to be highly variable between individuals ranging between complete or full recovery, to partial recovery or no recovery, with residual immunologically driven long-term biochemical effects (Holmes et al. 2021). It was also notable that the non-hospitalised mildly affected patients at 3-month follow-up also included some new phenotypic changes not observed in the controls or the acute phase patients. This included elevated plasma 3-indole acetic acid, a microbial product of tryptophan metabolism, indicating a possible microbiome activity shift which would not unsurprising given the complex immunological interactions at all stages of the disease. The significance of this observation remains to be determined. Phenoreversion appears to be a complex and variable process. Thus, the individual metabolic parameters used as Phenoreversion metrics can be substantially reversed or not reversed within the whole post-acute phase population cohort, and it is inevitable that some of these changes will impact on long-term disease risks for the affected individuals (Holmes et al. 2021). Even mildly symptomatic, non-hospitalized patients can show multiple symptoms months after the acute phase of the disease including chronic fatigue, joint pain anosmia and a multitude of neurological symptoms (Nalbandian et al. 2021). There are systematic metabolic differences between asymptomatic and symptomatic post COVID-19 patients. Given the number of affected individuals worldwide (currently approaching 170 million), the possibility that the long-term effects of COVID-19 on individuals and populations may be as great a healthcare and economic burden as the acute phase of the disease witnessed so far. Thus, it is essential to perform large-scale metabolic and clinical follow-up studies in affected groups to assess the prevalence of PACS and to assess individuals for metabolic effects that with help in the management of patients and the possible mitigation of the long-term symptoms.

Despite the rapid expansion of knowledge about the virus, there is still so much unknown about the COVID-19 disease and PACS processes in different populations, and with the steady evolution of new SARS CoV-2 variants we can also expect an expanded repertoire of biological properties of the virus which may present new medical challenges. An important future determinant of the pandemic outcomes will be the ability and agility to adapt healthcare policy to changes in the virus structure and biological properties. Metabolic phenotyping has previously been shown to be exceptionally effective at measuring and monitoring systemic pathological processes in multiple disease states including COVID-19, and so should become a vital tool for the molecular investigations and large-scale screening that are now ongoing round the world as part of the ongoing fight against the disease in “*The COVID-19 War”.*
